# Role of MicroRNA-155 as a Potential Biomarker for Allergic Rhinitis in Children

**DOI:** 10.1155/2021/5554461

**Published:** 2021-06-10

**Authors:** Noha M. Hammad, Fedaa Nabil, Eman M. Elbehedy, Randa Sedeek, Magdy I. Gouda, Mohamed A. Arafa, Saffaa M. Elalawi, Alia A. El Shahawy

**Affiliations:** ^1^Medical Microbiology and Immunology Department, Faculty of Medicine, Zagazig University, Zagazig, Egypt; ^2^Otorhinolaryngology, Head and Neck Surgery Department, Faculty of Medicine, Zagazig University, Zagazig, Egypt; ^3^Pediatrics Department, Faculty of Medicine, Zagazig University, Zagazig, Egypt; ^4^Clinical Pathology Department, Faculty of Medicine, Zagazig University, Zagazig, Egypt

## Abstract

**Background:**

Allergic rhinitis (AR) is an inflammatory state categorized by a disturbance of immunoregulatory mechanisms. MicroRNA-155 (miRNA-155) has an essential role in regulating gene expression and can mediate the allergic TH2 process.

**Objective:**

In this study, we aimed to evaluate the role of miR-155 as a biomarker in AR and correlate its level with the total nasal symptom score (TNSS) and the levels of serum interleukin-4 (IL-4).

**Methods:**

This study included 90 children: 45 with pollen-induced AR and 45 healthy controls. Serum miR-155 expression levels were measured using quantitative real-time PCR. Human IL-4 ELIZA kits were used for the semiquantitative detection of the serum levels of IL-4. Receiver operating characteristic (ROC) curves were used to determine the best cutoff values for the studied parameters for the diagnosis of AR.

**Results:**

The demographic characteristics of the two groups were matched with respect to age and sex. The AR case group included 23 (51.1%) males and 22 (48.9%) females, while the control group included 24 (53.3%) males and 21 (46.7%) females. The miR-155 level was increased in the serum of children with pollen-induced AR compared with controls (mean difference = 2.8, *p* < 0.001). A significant positive correlation between the serum expression level of miR-155 and TNSS in children with AR was detected (*r* = 0.494, *p* < 0.001). However, no significant correlation was identified between the expression of miR-155 and that of IL-4. At a cutoff value of 1.09, the sensitivity of miR-155 as a biomarker for AR was 100%, and the specificity was 71.1%.

**Conclusion:**

MiR-155 expression levels were elevated in the serum of AR children. Therefore, miR-155 could be used as a biomarker in AR diagnosis.

## 1. Introduction

Allergic rhinitis (AR) is the most common type of noninfectious chronic rhinitis with a significant world-wide prevalence, affecting 40% of the population [1]. Symptoms of AR include rhinorrhoea, sneezing, nasal itching, and congestion due to inﬂammation of the epithelium lining of the nose after exposure to allergens such as mites, insects, pollens, tobacco smoke, and aspirin or other nonsteroidal anti-inﬂammatory drugs [2]. The symptoms of AR may cause sleep disturbance, depression, fatigue, and decreased attention, impairing the quality of life and productivity of work [3].

Numerous inflammatory cells are responsible for the pathogenesis of AR, including mast cells, T cells, B cells, eosinophils, and macrophages that infiltrate the mucosal lining of the nose and throat upon exposure to an allergen. The cascade starts with the infiltration of *T*-helper-2 (Th2) cells into the nasal mucosa of allergic individuals and the release of a specific set of cytokines, including interleukin IL-3, IL-4, IL-5, and IL-13. These cytokines induce the production of immunoglobulin *E* (IgE) by plasma cells followed by the production of histamine and leukotrienes in the early phase of the immune response [4, 5]. The late-phase immune response results in recurrent AR symptoms and may be persistent [2].

The current treatment of AR is based on the alleviation of symptoms and mainly involves the use of steroids and antihistamines. Although the pathogenesis of AR is mainly immunological, the application of specific immunotherapy is still limited [1, 6]. Therefore, studying the pathogenesis of AR is important for the development of agents for AR treatment.

MicroRNAs (miRNAs) are short single-stranded (18–24 nucleotides) noncoding RNAs which play a major role in the regulation of gene expression by binding the 3′-untranslated site on the target messenger RNA (mRNA) to either induce RNA degradation or block protein synthesis [7].

The role of miRNAs has been confirmed in many inflammatory disorders, including allergic diseases, through alternating Th1/Th2 polarization and stimulating chronic epithelium inflammation by the activation of innate immune cells [8]. Circulating miRNAs could, therefore, be used as biomarkers owing to their role in allergic disease dysregulation and their relative stability compared with mRNAs [9].

Several studies have confirmed the role of miRNA-155 (miR-155) in the regulation of Th2 activation in response to allergen-induced eosinophilic airway inflammation [10]. Zech et al. (2015) confirmed that high levels of miR-155 expression were observed in the lungs of ovalbumin-challenged mice compared with control mice [11]. Karam and Abd Elrahman (2019) revealed that the expression level of miR-155 was significantly higher in the serum samples of asthmatic patients compared to healthy controls [12].

MiRNA-155 is thought to be involved in the pathogenesis of AR since its expression is upregulated in multiple immune cell lineages including T cells, B lymphocytes, mast cells, fibroblasts, macrophages, and dendritic cells [13–15]. The role of miR-155 in AR has been confirmed in studies by Suojalehto et al. (2013) [16], Zhu et al. (2019) [17], and Zhu et al. [18].

Furthermore, cytotoxic T-lymphocyte antigen 4 (CTLA-4) that binds to the CD28 ligand and blocks the activation of effector T lymphocytes is a possible mRNA target of miR-155 [19]. The increased expression of miR-155 and decreased expression of CTLA-4 in T lymphocytes were demonstrated in a study conducted by Sonkoly et al. and suggested that miR-155 inhibition of CTLA-4 corresponds to the stimulation of effector T cells in the skin of patients with atopic dermatitis [20].

In addition, miR-155 plays a critical role as a potential target for patients with allergic diseases, and the modulation of miR-155 expression could be considered as a potential treatment for human diseases [21]. In this work, we aimed to assess the expression of miR-155 in children with AR and correlate its level with those of TNSS and serum IL-4.

## 2. Materials and Methods

### 2.1. Study Participants

This case-control study was conducted on 45 children with AR attending the outpatient allergy unit at the Faculty of Medicine, Zagazig University, as well as 45 age-matched healthy children without any family history of allergy who served as the control group during the period from September 2019 to March 2020. The study was conducted in accordance with the Declaration of Helsinki and was approved by the Institutional Review Board, Faculty of Medicine, Zagazig University. Written informed consent was obtained from the parents of each participant, and verbal assent was taken from the children included in the study, after explaining the nature of the investigation as well as the purpose of the study. We included patients who were allergic to grass pollen as confirmed by medical history, general and local examination, and specific IgE assay. Children who had active upper respiratory tract infections or bronchial asthma within four weeks before sample collection were excluded.

### 2.2. Total Nasal Symptom Score (TNSS)

We assessed the nasal symptoms of the study participants using a TNSS. This score was calculated by the sum of scores for each of nasal congestion, sneezing, nasal itching, and rhinorrhea at each time point, using a four-point scale (0–3), where a score of 0 indicates the absence of symptoms, a score of 1 indicates mild tolerable symptoms, a score of 2 indicates annoying but tolerable symptoms, and a score of 3 is reserved for severe symptoms which restrict daily activity. The TNSS was calculated by adding the score for each of the symptoms, to produce a total out of 12 [22].

### 2.3. Serum Samples

A 5 ml sample of peripheral blood was withdrawn from each subject via venipuncture under complete aseptic conditions and centrifuged at 1000 xg for 10 minutes to separate the serum for specific IgE and IL-4 assays. Separated serum samples were centrifuged at 16,000 x g for 10 minutes. Samples were stored at –80°C until the extraction of small RNAs. The laboratory tests were performed at the Medical Scientific Research Center, Faculty of Medicine, Zagazig University.

### 2.4. Measurement of Serum-Specific IgE

Quantitative measurement of specific IgE was performed using immune blot assay against aeroallergens. According to the manufacturer's instructions, anti-goat IgG was used as a positive control (Allergy Screen Panel 2A EGY, MEDIWISS analytic GmbH, Underinger, Germany). Serum-specific IgE was analyzed by Rapid Reader (Improvio, Germany). The result was shown as IU/ml, with a detection limit of 0.35 kAU/L. Valid rest was considered when the positive control IgE was more than 3.5 IU/ml.

### 2.5. Quantitative Measurement of Human Serum IL-4

Human IL-4 ELIZA kits (Sunlong Biotech, Hangzhou, China) were used for semiquantitative detection of serum levels of IL-4 in the samples, with the analysis performed according to the manufacturer's instructions.

### 2.6. Quantitative Real-Time Polymerase Chain Reaction (qRT-PCR) and miRNA Assay

Isolation of miRNA was achieved by means of miRNeasy Serum/Plasma Kits (Qiagen, Germany) according to the manufacturer's instructions. The “miScript II RT Kit” (Qiagen, Germany) was used to perform the reverse transcription reaction. Areverse transcription master mix was prepared by adding the following ingredients to produce a total volume of 20 *μ*l: 4 *μ*l 5x miScript HiSpec Buffer, 2 *μ*l 10x miScript Nucleics Mix, 2 *μ*l miScript Reverse Transcriptase Mix, 2 *μ*l Template RNA, and 10 *μ*l RNase-free water. The reaction mixture was incubated for 60 min at 37°C. Then, it was incubated for 5 min at 95°C on a “Rocker Sahara 320 dry bath heat block” to inactivate miScript RT and then placed immediately on ice and stored at −80^o^c.

The miScript SYBR Green PCR Kits (Qiagen, Germany) were used for real-time PCR quantification of mature miR-155 using target-specific miScript Primer Assays. Two separate tubes were prepared for each sample, one for miR-155 and the other for the human RNU6B (RNU6-2) as a normalization gene for later assessment of miR-155 expression. The reaction mixture was prepared as follows according to the kit manufacturer recommendations to reach a total volume of 18 *μ*l: 10 *μ*l 2x QuantiTect SYBR Green PCR Master Mix, 2 *μ*l 10x miScript Universal Primer, 2 *μ*l 10x miScript Primer Assay (miR-155 or RNU-6), and 4 *μ*l RNase-free water. Then, 2 *μ*l of template cDNA was added to reach a final reaction volume of 20 *μ*l.

Real-time PCR was performed using the following cycling conditions: an initial activation step of 15 min at 95^o^c to activate HotStar Taq DNA Polymerase, followed by 40 cycles of denaturation at 95°C for 15 sec, then annealing at 55°C for 30 sec, and extension at 70°C for 30 sec. We used the “Stratagene Mx3005P” platform (Agilent Technologies, USA) to determine the threshold cycle (Ct) value. Fold changes of miR-155 expression were estimated using the 2^−ΔΔCT^ method [23].

### 2.7. Statistical Analysis

Statistical analyses were implemented using the statistical software program, SPSS, for Windows version 25.0 (SPSS; Chicago, IL, USA) and MedCalc (MedCalc 10 Software, Ostend, Belgium). Continuous variables were presented as mean, standard deviation (SD), and range. Categorical variables were described as frequency and percentages. Comparisons between quantitative variables were performed using independent sample *t*-testsfor parametric data or Mann–Whitney tests for nonparametric data. Chi-square tests were used to compare categorical data. Correlations between the expression levels of miR-155 and serum levels of IL-4, TNSS score, specific IgE, and age in AR cases were assassed using Spearman's correlation coefﬁcient. We used receiver operating characteristic (ROC) curves to determine the best cutoff for the studied parameters in the diagnosis of AR. *P* values of <0.05 were considered to be statistically significant.

## 3. Results

### 3.1. Demographic Characteristics

The present study included 45 children with AR and 45 healthy controls. The demographic characteristics of the two groups are presented in Table 1. Both groups were matched with respect to age and sex. The AR patient group included 23 (51.1%) males and 22 (48.9%) females, while the control group included 24 (53.3%) males and 21 (46.7%) females. For the AR group, the mean TNSS was 8.8 (±1.47), while the mean specific IgE titer was 14.4 (±5.84).

### 3.2. Measurement of miRNA-155 and IL-4

**Figure 1:**
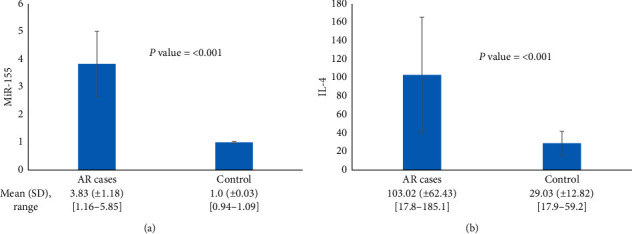
Comparison between the studied groups regarding (a) miR-155 and (b) IL-4 serum levels. AR: allergic rhinitis and SD: standard deviation.

MiR-155 expression levels were significantly higher in AR children than in control subjects (mean difference = 2.8, 95% CI [3.2 to 2.5]), Figure 1(a). IL-4 serum levels were also significantly higher in AR children than in control children (mean difference = 74.01, 95% CI [92.88 to 55.12]), Figure 1(b).

**Figure 2:**
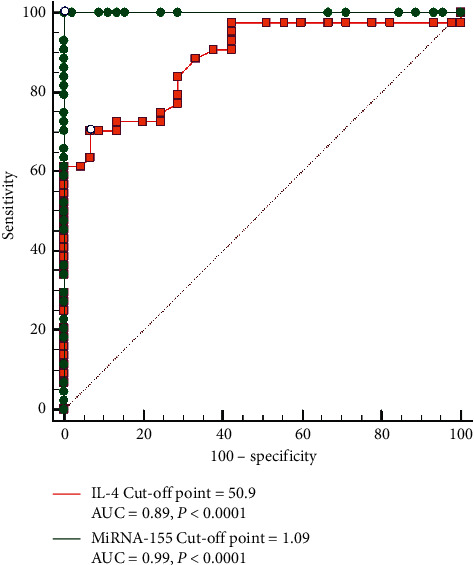
ROC curve showing the performance of miRNA-155 and IL-4 in the diagnosis of allergic rhinitis.

ROC curve analysis was performed to assess the values of miR-155 and IL-4 levels as biomarkers for AR (Figure 2). The best cutoff of miR-155 in the diagnosis of AR was ≥1.09 which produced an area under the curve (AUC) of 0.99 (95% CI [0.96 to 1.0]), sensitivity 100%, specificity 71.1%, positive predictive value (PPV) 77.6%, and negative predictive value (NPV) 100%. The best cutoff value of IL-4 in the diagnosis of AR was 50.9, with an AUC of 0.89 (95% CI [0.79 to 0.94]), sensitivity 69%, specificity 93.3%, PPV 91.2%, and NPV 75%.

### 3.3. Correlation between miR-155 and IL-4 Levels, Specific IgE, and TNSS in AR Children

**Figure 3:**
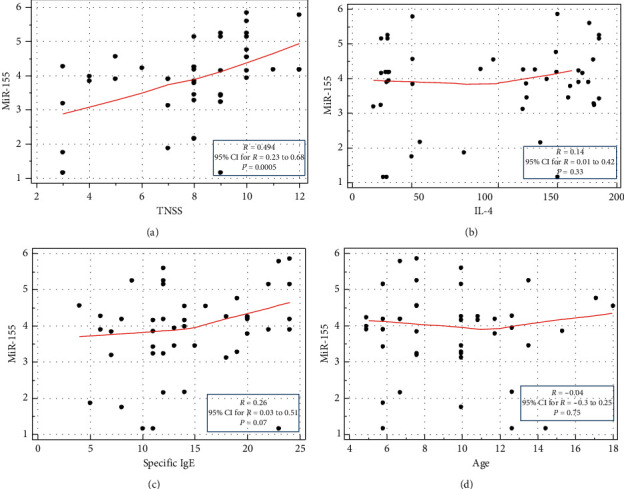
Correlations between miR-155 and (a) TNSS, (b) IL-4, (c) specific IgE, and (d) age among AR patients.

This analysis revealed a statistically significant positive correlation between the expression levels of miR-155 and those of TNSS (*r* = 0.494, *P* < 0.001, Figure 3(a)). There was no significant correlation between miR-155 and IL-4 (*r* = 0.14, *P*=0.33), specific IgE (*r* = 0.26, *P*=0.07), and age (*r* = 0.04, *P*=0.75) as seen in Figures 3(b)–3(d).

## 4. Discussion

Recent studies have indicated that miRNAs are involved in the pathogenesis of AR in many ways, including the regulation of mast cells, eosinophils, and *T*-helper cell (Th) 1/Th2 disproportion [17, 24]. Reports of allergic illnesses have indicated that miR-155 has an essential role in promoting inflammation and Th2 immunity [25]. As reported by Malmhäll and colleagues (2014), miR-155 knockout mice showed a decrease in Th2 cell numbers and airway cytokines (IL-4, IL-5, and IL-13), an observation which confirms that miR-155 contributes to the regulation of allergic airway inflammation [10].

The present study demonstrated that miR-155 was significantly upregulated in the serum of AR children compared to healthy controls. This ﬁnding is in agreement with that published by Suojalehto et al., who showed that the expression level of miR-155 was amplified in the nasal tissue of patients with AR [16]. Zhu et al. reported that miR-155 expression was elevated in the nasal mucosa of AR patients and a positive linear correlation was found between the percentage of innate lymphoid cells (ILCs) and miR-155 expression [17]. However, these studies measured miR-155 in nasal tissue, not in the serum of AR patients. A recently published animal study showed that miR-155 upregulation significantly increased the rates of nasal rubbing and sneezing and levels of IL-4, IL-5, IL-9, and IL-13. Also, pathological alterations were exacerbated after the intranasal administration with miR-155 agomir and were improved after miR-155 antagomir administration [18].

With respect to other allergic conditions, Elkashef et al. revealed that the expression of miR-155 was significantly higher in the serum samples of asthmatic patients compared to healthy controls [26]. Furthermore, Sonkoly et al. showed that miR-155 upregulated in patients with atopic dermatitis was predominantly expressed in infiltrating immune cells [20]. On the other hand, Panganiban et al. reported a significant downregulation of miR-155 in patients with AR, but significant upregulation in asthmatic patients compared to healthy individuals [27]. Low expression levels of miR-155 were also observed by Malmhall et al. in cell-free sputum supernatants from an allergic asthmatic group compared to healthy controls [28]. This difference might be explained by differences in locality and genetic differences between the patients.

On the contrary, miR-155 has been identified, in initial studies, as a Th2 suppressor. High expression of miR-155 in CD4-positive cells has been shown to augment Th1 response after stimulation with IFN-gamma. However, its suppression has induced Th2 differentiation in response to IL-4 [29, 30]. The present studies have produced new evidence that miRNA-155 plays an essential role in the promotion, rather than the suppression, of Th2 pathways [26]. The present analysis showed that IL-4 levels were significantly elevated in patients with AR. Nevertheless, there was a positive but nonsignificant correlation between miR-155 and IL-4 (*P*=0.33). These results are in agreement with those of Hasegawa et al., who detected a significant increase in IL-4 in allergic individuals [31]. Elkashef et al. also observed a nonsignificant correlation between the expression of miR-155 and IL-4 serum levels in asthmatic patients [26]. Nevertheless, Suojalehto et al. observed a weak positive correlation between miR-155 and Th2 cytokine levels in asthmatic patients [32]. Furthermore, Johansson et al. proposed that miR-155 acts as a positive regulator in allergic inflammation mediated by type 2 ILCs and IL-33. MiR-155 knockout mice exhibited deficient IL-33-mediated allergic inflammatory signalling and type 2 ILCs expansion [33]. Taken together, it seems that not only TH2 immune responses but also other immune mechanisms at local and systemic levels should be investigated to explain their associations and influence on miR-155 expression.

In the current study, a significant positive correlation was detected between the miR-155 level and TNSS in children with AR. On the contrary, Liu et al. revealed that decreased regulatory T-cell-derived miR-155 was correlated with reduced Tregs percentage in children with AR. Negative correlations between the TNSS and Treg-derived miR-155 levels were also observed [34]. These differences could be explained by the difference in the miR-155 source as we had assessed serum miR-155, not intracellular miR-155. In addition, a weak positive correlation between miR-155 and IgE was also observed by Suojalehto et al. [32]. Our results, with a larger sample size, revealed no significant correlation between the levels of miR-155 and those of IgE.

ROC analysis demonstrated that the best cutoff for miR-155 in the diagnosis of AR was ≥1.09 with 100% sensitivity and 71.1% specificity. Our cutoff value was in agreement with the results of ElKashef et al., who concluded that the best cutoff of miR-155 in asthma diagnosis was 1.96, with 100% sensitivity and 100% specificity for the diagnosis of eosinophilic asthma [26].

Furthermore, Karam and Abd Elrahman stated that miR-155 could help in asthma diagnosis, disease severity prediction, and the likelihood of response to therapy [12].

One of the strengths of this study is the relatively large sample size. The post hoc analysis was performed for power calculation and revealed 100% power in this study. The main limitation of this study was that we were unable to measure the correlation between miR-155 and other Th2 cytokines for financial reasons. Therefore, further investigation in this direction is highly recommended.

## 5. Conclusions

MiR-155 expression levels were observed to be increased in the serum of children with AR compared with those of healthy children. Larger and more comprehensive studies are still needed to verify whether miR-155 can be used as a diagnostic marker for AR.

## Figures and Tables

**Table 1:** Demographic data of the cases and controls.

	AR cases (*n* = 45)	Control (*n* = 45)	*P* value
*Age (year)*			
Mean (±SD), [range]	9.07 (±3.11), [5–18]	9.64 (±3.29), [5–18]	0.395

*Gender, n (%)*			
Female	22 (48.9%)	21 (46.7%)	0.833
Male	23 (51.1%)	24 (53.3%)	

Body mass index	27.31 ± 5.64	25.46 ± 4.92	0.101

Specific IgE	—	—	—
Mean (±SD), [range]	14.4 (±5.84), [4–24]	—	—

TNSS	—	—	—
Mean (±SD), [range]	8.8 (±1.47), [4–12]	—	—

AR: allergic rhinitis, SD: standard deviation, TNSS: total nasal symptom score

## Data Availability

The data that support the findings of this study are available from the corresponding author upon reasonable request.
